# Subjective reports of physical activity levels and sedentary time prior to hospital admission can predict utilization of hospital care and all-cause mortality among patients with cardiovascular disease

**DOI:** 10.1177/1474515120921986

**Published:** 2020-05-05

**Authors:** Amanda Ek, Lena V Kallings, Mattias Ekström, Mats Börjesson, Örjan Ekblom

**Affiliations:** 1 The Åstrand Laboratory of Work Physiology, The Swedish School of Sport and Health Sciences, Sweden; 2 Functional Area Occupational Therapy & Physiotherapy, Allied Health Professionals Function, Karolinska University Hospital, Sweden; 3 Unit of General Practice, Department of Public Health and Caring Sciences, Uppsala University, Sweden; 4 Department of Clinical Sciences, Danderyd Hospital, Division of Cardiovascular Medicine, Sweden; 5 Department of Medicine, Solna, Karolinska Institutet, Sweden; 6 Department of Neuroscience and Physiology, Sahlgrenska Academy & Sahlgrenska University Hospital/Ostra, Sweden; 7 Centre for Health and Performance, Department of Food, Nutrition and Sport Science, University of Gothenburg, Sweden

**Keywords:** Hospitalization, physical exercise, sedentary behaviour, survival

## Abstract

**Background:**

In prevention, sedentary behaviour and physical activity have been associated with risk of cardiovascular disease and mortality. Less is known about associations with utilization of hospital care.

**Aim:**

To investigate whether physical activity level and sedentary behaviour prior to cardiac ward admission can predict utilization of hospital care and mortality among patients with cardiovascular disease.

**Methods:**

Longitudinal observational study including 1148 patients admitted and treated in cardiac wards in two hospitals. Subjective reports of physical activity levels and sedentary time prior to admission were collected during inpatient care and categorized as low, medium or high. The associations between physical activity level and sedentary time with hospital stay, readmission and mortality were analysed using linear, logistic and Cox regressions.

**Results:**

Median hospital stay was 2.1 days. One higher step in the physical activity level, or lower sedentary time, was related to an approximately 0.9 days shorter hospital stay. Sixty per cent of patients were readmitted to hospital. The risk of being readmitted was lower for individuals reporting high physical activity and low sedentary time (odds ratios ranging between 0.44 and 0.91). A total of 200 deaths occurred during the study. Mortality was lower among those with high and medium physical activity levels and low sedentary time (hazard ratios ranging between 0.36 and 0.90).

**Conclusion:**

Both physical activity level and sedentary time during the period preceding hospitalization for cardiac events were predictors of hospital utilization and mortality. This highlights the prognostic value of assessing patients’ physical activity and sedentary behaviour.

## Introduction

Physical activity is defined as ‘any bodily movement produced by the contraction of skeletal muscle that increases energy expenditure at basal level’. Exercise is a subcategory of physical activity and is defined as ‘planned, structured, repetitive and purposive in the sense that the improvement or maintenance of one or more components of physical fitness is the objective’.^1^

As early as 1966, Morris et al. published a study showing lower incidence of ischaemic heart disease in bus conductors compared with drivers.^2^ More recent studies have explained this phenomenon by showing that both regular physical activity and low levels of sedentary behaviour are associated with reduced risk of cardiovascular disease (CVD) and mortality.^3–5^ Additionally, regular physical activity is universally recommended for prevention of CVD in international guidelines.^6^,^7^ However, a large part of the adult population has been reported as having an insufficiently high physical activity level and/or a high sedentary behaviour level.^3^

Routine assessment of physical activity in healthcare as a ‘vital sign’ has been proposed by the American College of Sports Medicine.^7^ Epidemiological studies indicate that individuals with higher levels of regular physical activity have fewer inpatient and outpatient care visits,^8^,^9^ shorter inpatient duration^8^ and a lower incidence of circulatory disease events^10–12^ compared with individuals with low levels of regular physical activity. Among patients with ischaemic heart disease (IHD) or heart failure, a high level of physical activity or low level of sedentary behaviour decreases the risk of readmission.^13^,^14^ For mortality, there was no risk reduction with increased physical activity level in short-term follow-up (≤12 months) among patients with heart failure.^13^ However, a risk reduction was seen in long-term follow-up (24 months) and among patients with IHD who self-reported a higher physical activity level.^14^,^15^

To our knowledge, there are no available studies focusing on duration of inpatient care specifically for patients with CVD. In addition, the predictive value of physical activity level and sedentary time (SED) at admission, on hospital utilization and on survival among individuals with other types of CVD, for example, cardiac arrhythmias, valvular heart disease and inflammatory heart diseases (endocarditis, pericarditis and myocarditis), is not fully known.

Importantly, there is a weak association between physical activity level and SED, for example, individuals with a high level of high intensity physical activity might also spend a lot of time being sedentary.^5^ This highlights the importance of measuring physical activity level and SED separately.

There is a need for increased knowledge on whether physical activity level and SED prior to cardiac ward admission may predict hospital utilization and mortality among patients with CVD. The clinical implications are important as organizations advocate routine physical activity assessment on admission to hospital.^6^,^7^

Therefore, this study aims to investigate whether self-reported physical activity levels and SED prior to cardiac ward admission can predict utilization of hospital care (inpatient duration and readmission) and mortality among patients with CVD.

## Methods

### Study population

This longitudinal observational study included patients treated on cardiac wards at two hospitals in Stockholm, Sweden. Inclusion criteria were: patients who were subsequently discharged alive, 18 years or older, had a Swedish personal identification number and had been hospitalized for at least one day during weekdays between 1 September 2015 and 30 April 2016. Patients with cognitive dysfunction (e.g. dementia) or poor Swedish language skills were excluded in order to decrease the risk of misinterpretation of the questions. Additionally, patients with acute life-threatening CVD (severe pulmonary oedema) were excluded due to ethical reasons. Finally, in the analyses, individuals that did not have complete data were excluded. The Regional Ethics Board in Stockholm, Sweden approved the study (Dnr: 2016/1057-31/5) and the investigation conformed to the principles outlined in the Declaration of Helsinki.^16^

### Data

**Figure 1.**
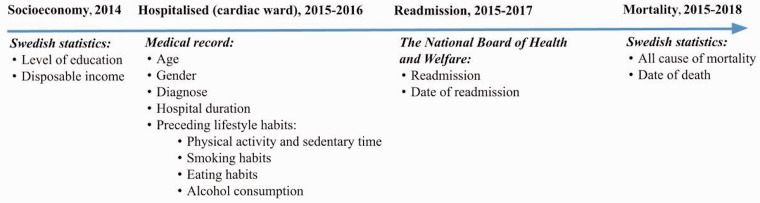
Flowchart of data collection describing when and where data were extracted from.

Patient data were extracted from several databases. Information on when and from where data were extracted is described in Figure 1. The outcome measures were hospital utilization (inpatient duration, readmission) and all-cause mortality. Duration of hospitalization (in days) was collected from the patient’s medical records. Data on readmission to any hospital was obtained from the National Board of Health and Welfare and recorded until 31 December 2017. Mortality was measured as the survival time after end of treatment on the cardiac ward, which was obtained from official statistics (Statistics Sweden) with data registered until 26 November 2018.

While on the cardiac ward, patients used a questionnaire (Supplementary Material 1 online) to self-rate their physical activity level and SED prior to admission. The questionnaire contained validated questions recommended by The Swedish National Board of Health and Welfare^17–19^ and included three different measures of physical activity (physical exercise, everyday physical activity, index of total physical activity level) as well as SED:

1. *Physical exercise*. ‘During a regular week, how much time do you spend exercising on a level that makes you short winded, for example running, fitness class, or ball games?’ The patient selected six fixed answers, which were categorized into three groups: low (0 min/week), medium (1–60 min/week) and high (≥60 min/week). Stratification was performed to create groups with meaningful sizes.
2. *Everyday physical activity*. ‘During a regular week, how much time are you physically active in ways that are not exercise, for example walks, bicycling, or gardening? Add together all activities lasting at least 10 min.’ Seven fixed answers were selected and categorized into three groups: low (≤29 min/week), medium (30–149 min/week) and high (≥150 min/week). The highest category corresponded to the internationally recommended level of physical activity for health, that is, at least 150 min of physical activity per week, of at least moderate intensity.
3. The questions regarding physical exercise and everyday physical activity formed an index (3–19 points) of total physical activity level. This was obtained by multiplying the category of exercise (one to six) by two (to account for a proposed higher intensity) and then adding the category of everyday physical activity (one to seven).^17^ The index of total physical activity level was categorized into three groups: low (3–6 points), medium (7–9 points) and high (10–19 points). The cut-offs were made in order to create three groups of approximately equal size.
4. SED was measured by the question: ‘How much time do you sit during a normal day, excluding sleep?’^18^ There were seven answer options, which were later grouped into three categories, low (<6 h), medium (7–9 h) and high (≥10 h). The cut-off points were based on a meta-analysis suggesting that the risk of all-cause mortality increases if adults sit for a total of ≥7 h.^20^

Covariates associated with the risk of cardiovascular disease and all-cause mortality^6^,^21–25^ were obtained from medical records from the current hospitalization. These included: gender, age, diagnosis group (IHD, heart failure, cardiac arrhythmia, valvular heart disease, inflammatory heart diseases) and lifestyle habits (smoking, eating habits and use of alcohol). Lifestyle habits pre-admission were assessed using a questionnaire (Supplement 1) based on recommendations from the Swedish National Board of Health and Welfare.^19^ Smoking habits were categorized as ‘never smoked’, ‘stopped smoking more than six months ago’ and ‘current smoker or stopped within the last six months’. To assess eating habits, the participants answered questions on how often they ate vegetables, fruits/berries, fish/shellfish and sweets. The answers were divided into four categories (0–3). An eating index was developed based on the sum of those four categories: ‘0–4, considerably unhealthy eating habits’, ‘5–8, moderately healthy eating habits’, ‘≥9, follows healthy eating recommendations’. Hazardous use of alcohol was assessed by the question ‘How often do you drink 4 (women)/5 (men) units of alcohol on a single occasion?’ One alcohol unit is equivalent to 10 ml of pure alcohol. There were six categorical answers. One or more occasions per month was considered hazardous use. In addition, information regarding educational level and disposable income was collected from Statistics Sweden. Educational level was categorized as secondary school (nine years total), college (12 years total) or higher vocational education or university (>12 years). Disposable income was calculated as the sum of income minus final tax. The median disposable annual income in Sweden in 2014 was SEK338,400.^26^ The disposable income was divided into three groups: low, middle and high. Disposable income of < 60% of the Swedish median was categorized as low while a disposable income of twice the median was categorized as high.

### Statistics

Descriptive demographics and clinical characteristics were presented as frequencies and relative frequencies or as medians with interquartile ranges (IQRs). Differences between included versus excluded patients, included patients versus drop-outs, patients with and without readmission, and survivors versus non-survivors were examined using the Chi-square test for categorical data and the Mann–Whitney *U* test for continuous non-parametric data.

Correlations between physical activity level, SED and inpatient duration were assessed using Spearman’s partial rank order correlation. When a significant correlation was found, the data were analysed further using multiple linear regression including age, gender, diagnosis group, educational level, disposable income, smoking status, alcohol consumption and eating habits. Residuals of the natural logarithm of inpatient duration were found to be normally distributed.

To explore the association between physical activity level, SED and risk of readmission, logistic regressions were used. Odds ratios (ORs) for physical activity and sedentary strata (low, medium and high) were analysed using three models: unadjusted, adjusted for age and gender, and fully adjusted. In the fully adjusted models, age, gender, diagnosis group, educational level, disposable income, smoking status, alcohol consumption and eating habits were included.

Before mortality hazard ratios and their 95% confidence intervals were calculated, the proportionality assumption was checked using the Schoenfeld residuals method. Since a weak significance for all physical activity and SED categories was noted, an interaction term (time × physical activity and SED strata) was used in all analyses using a Cox regression with a time dependent covariate module. Hazard ratios for physical activity and sedentary strata (low, medium and high) were analysed using the same three models as with the logistic regressions. In order to explore whether there was a difference between the diagnosis groups regarding the effect of physical activity level and SED, an interaction term was added to our fully adjusted linear, logistic and Cox regression.

Hazard ratios and ORs were considered statistically significant if the 95% confidence interval did not include 1. Formal interaction analyses for hazard ratios and ORs between physical activity strata (medium and high) were performed as proposed by Bland and Altman.^27^ All statistical analyses were performed using the SPSS 24.0 software (IBM Corp., Armonk, New York, USA).

## Results

**Figure 2.**
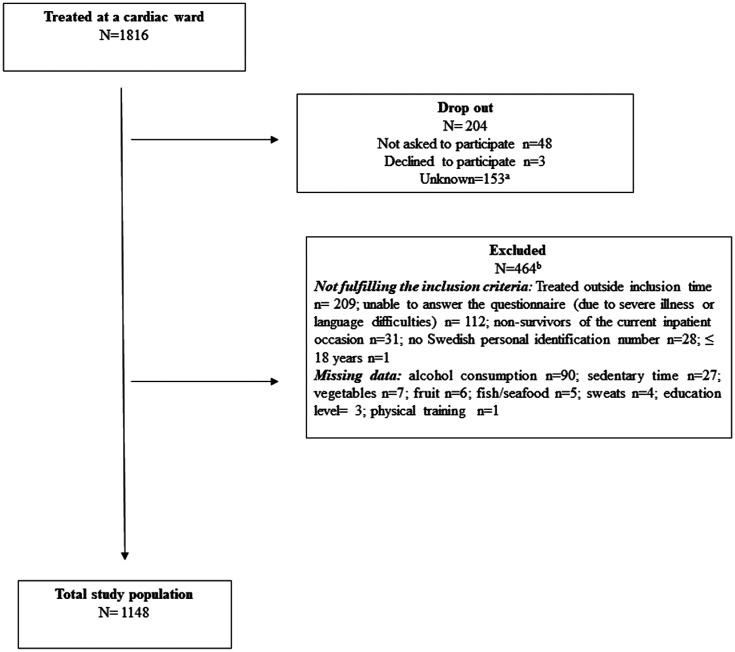
Flowchart of recruitment of study population. ^a^Due to structural reasons at one hospital, it was not possible to obtain information on which patients were not asked to participate, or declined, respectively. ^b^Excluded due to not fulfilling one or several inclusion criteria.

A total of 1816 individuals were treated on cardiac wards during the inclusion period. Of those, 204 individuals were not asked to participate or did not want to participate (drop-out). Further, 464 individuals did not fulfil the inclusion criteria. This gave a total study population of 1148 individuals (Figure 2), where 61% were men and median age was 70 years (Table 1). Approximately half the study population stated that they did not participate in any physical exercise pre-admission, and one-fifth stated that they spent < 30 min in everyday physical activity during an average week. In addition, one-fourth were sitting ≥10 h a day. A minority were smokers (15%), approximately one-fourth followed the recommendations for healthy eating habits and one-fifth were categorized as having hazardous use of alcohol.

**Table 1.** Patient baseline characteristics in total group and divided by individuals with non-readmission versus readmission and survivors versus non-survivors.

	Total group*N* = 1148 (%)	Non-readmission*n* = 326 (%)	Readmission*n* = 822 (%)	Survivors*n* = 948 (%)	Non-survivors*n* = 200 (%)
**Gender, male**	703 (61)	269 (59)	434 (63)	580 (61)	123 (62)
**Age, median (IQR)**	70 (IQR 19)	66 (IQR 19)^a^	72 (IQR 20)	68 (IQR 19)	79 (IQR 15)^b^
**Educational level**		^a^			
Secondary school: ≤9 years	294 (26)	108 (24)	186 (27)	220 (23)	74 (37)^b^
College: 10–12 years	465 (41)	172 (38)	293 (42)	391 (41)	74 (37)
Higher vocational education/university: > 12 years	389 (34)	176 (39)	213 (31)	337 (36)	52 (26)
**Disposable income**		^a^			^b^
Low: ≤SEK203,040	600 (52)	218 (48)	382 (55)	457 (48)	143 (72)
Medium: SEK203,041–676,799	492 (43)	219 (48)	273 (40)	441 (47)	51 (26)
High: ≥SEK676,800	56 (5)	19 (4)	37 (5)	50 (5)	6 (3)
**Group of diagnosis**		^a^			^b^
Ischaemic heart disease	396 (34)	179 (39)	217 (31)	340 (36)	56 (28)
Heart failure	147 (13)	35 (8)	112 (16)	84 (9)	63 (32)
Arrhythmia	282 (25)	109 (24)	173 (25)	247 (26)	35 (18)
Valvular heart disease	108 (9)	28 (6)	80 (12)	84 (9)	24 (12)
Inflammatory heart diseases	59 (5)	25 (6)	34 (5)	53 (6)	6 (3)
Other	156 (14)	80 (18)	76 (11)	140 (15)	16 (8)
**Exercise, per week**		^a^			^b^
Low: 0 min	640 (56)	236 (52)	404 (58)	486 (51)	154 (77)
Medium: <60 min	298 (26)	119 (26)	179 (26)	266 (28)	32 (16)
High: ≥60 min	210 (18)	101 (22)	109 (16)	196 (21)	14 (7)
**Everyday physical activity, per week**		^a^			^b^
Low: ≤29 min	241 (21)	66 (15)	175 (25)	172 (18)	69 (35)
Medium: 30–149 min	500 (44)	202 (44)	298 (43)	419 (44)	81 (41)
High: ≥150 min	407 (35)	188 (46)	219 (32)	357 (38)	50 (25)
**Total physical activity level** ^c^		^a^			^b^
Low: 3–6 points	409 (36)	140 (31)	269 (39)	304 (32)	105 (53)
Medium: 7–9 points	382 (33)	154 (34)	228 (33)	316 (33)	66 (33)
High: ≥10	357 (31)	162 (36)	195 (28)	328 (35)	29 (15)
**Sedentary time, per day)**		^a^			^b^
High: ≥10 h	313 (27)	100 (22)	213 (31)	229 (24)	84 (42)
Medium: 7–9 h	275 (24)	125 (27)	150 (22)	226 (24)	49 (25)
Low: 0–6 h	560 (49)	231 (51)	329 (48)	493 (52)	67 (34)
**Smoking**					
Never smoked	466 (41)	189 (41)	277 (40)	389 (41)	77 (39)
Former smoker: >6 months	513 (45	193 (42)	320 (46)	411 (43)	102 (51)
Smoker, or stopped within the last six months	169 (15)	74 (16)	95 (14)	148 (16)	21 (11)
**Eating habits**					^b^
Considerable unhealthy eating habits: ≤4 points	181 (16)	76 (17)	105 (15)	150 (16)	31 (16)
Moderately health eating habits: 5–8 points	676 (59)	255 (56)	421 (61)	539 (57)	137 (69)
Follows the healthy eating recommendations: ≥9 points	291 (25)	125 (27)	166 (24)	259 (27)	32 (16)
**Hazardous use of alcohol**	216 (19)	92 (20)	124 (18)	195 (21)	21 (11)^b^

^a^Differences between individuals without and with readmission.

^b^Differences between survivors and non-survivors.

^c^Index of total physical activity level (the physical exercise and everyday physical activity question formed an index (3–19 points)).

There were differences in age and diagnosis between drop-out and included patients. The drop-out group was significantly older (73 (IQR 19) *vs*. 70 (IQR 20) years). In addition, fewer were diagnosed with heart failure (4.9% *vs*. 12.8%), inflammatory heart disease (0.5% *vs*. 5.1%) or valvular heart disease (3.9% *vs*. 9.4%) and they were more commonly diagnosed with other diseases (32.8% *vs*. 13.6%). There were no differences regarding gender or age between included and excluded patients. However, there was a significant difference between diagnosis groups, with more patients with valvular heart disease (9.4% *vs*. 4.4%) being included.

### Hospital stay

The median inpatient cardiac ward duration was 2.1 days (IQR 3); however, the hospital duration differed between different diagnosis groups (Table 2). There were bivariate correlations between inpatient duration and all physical activity and sedentary results. From the multiple linear regression, inpatient duration was 0.92, 0.91 and 0.91 days shorter for each higher category in physical exercise, everyday physical activity and total physical activity level, respectively. With one lower SED category, hospital duration was on average 0.92 days shorter (Supplement 2). Regarding inpatient duration, there was no difference in physical activity and SED for the different diagnosis groups.

**Table 2.** Inpatient cardiac ward duration (median with IQR) for different diagnosis groups.

	Days
Ischaemic heart disease	2.6 (IQR 2.7)
Heart failure	2.6 (IQR 3.6)
Arrhythmia	1.7 (IQR 1.7)
Valvular heart disease	3.31 (IQR 2.9)
Inflammatory heart diseases	2.7 (IQR 2.9)
Other	1.6 (IQR 1.9)

### Readmission

Baseline differences between patients with and without readmission are described in Table 1. Of the total number of patients, 692 individuals (60%) had at least one hospital readmission during the follow-up (median 338 days to first readmission or end of study). Table 3 describes ORs for different physical activity and SED levels. In unadjusted and adjusted analyses, the risk of being readmitted to hospital was lower for individuals reporting high and medium levels of everyday physical activity. For physical exercise, ORs were lower for individuals with high levels of exercise compared with individuals with low levels in the unadjusted model. For total physical activity level, individuals with high physical activity levels had lower ORs for readmission compared with individuals with low total physical activity levels in both the unadjusted and age and gender adjusted analyses. In unadjusted and adjusted analyses, individuals reporting medium or low SED had a decreased risk of readmission compared with those with a high level of SED. The relationship between everyday physical activity, exercise, total physical activity level, SED and risk of readmission did not differ between the diagnosis groups.

**Table 3.** Unadjusted and adjusted odds ratios (odds ratios with 95% confidence intervals) of readmission to hospital among patients diagnosed with cardiovascular disease with different level of physical activity and sedentary time.

	Unadjusted	Adjusted odds ratio^a^	Adjusted odds ratio^b^
**Everyday physical activity,** per week			
**Low**: ≤29 min	1, reference	1, reference	1, reference
**Medium**: 30–149 min	0.56 (0.40–0.78)	0.57 (0.41–0.80)	0.58 (0.41–0.82)
**High:** ≥150 min	0.44 (0.31–0.62)	0.47 (0.33–0.67)	0.48 (0.33–0.68)
**Physical exercise,** per week			
**Low**: 0 min	1, reference	1, reference	1, reference
**Medium**: <60 min	0.88 (0.66–1.17)	0.90 (0.67–1.20)	0.91 (0.68–1.23)
**High**: ≥60 min	0.63 (0.46–0.86)	0.80 (0.57–1.11)	0.83 (0.59–1.17)
**Index of total physical activity level**^c^, per week			
**Low**: 3–6 points	1, reference	1, reference	1, reference
**Medium**: 7–9 points	0.77 (0.58–1.03)	0.78 (0.58–1.04)	0.79 (0.59–1.06)
**High**: ≥10	0.62 (0.47–0.84)	0.73 (0.54–0.99)	0.75 (0.55–1.03)
**Sedentary time,** per day			
**High**: ≥10 h	1, reference	1, reference	1, reference
**Medium**: 7–9 h	0.67 (0.50–0.90)	0.69 (0.51–0.93)	0.69 (0.51–0.93)
**Low**: 0–6 h	0.56 (0.40–0.79)	0.57 (0.41–0.81)	0.58 (0.42–0.83)

^a^Adjusted for age and gender.

^b^Adjusted for age, disposable income, eating habits, educational level, gender, diagnosis group, hazardous use of alcohol and smoking status.

^c^The physical exercise and everyday physical activity question formed an index (3–19 points).

### All-cause mortality

**Figure 3.**
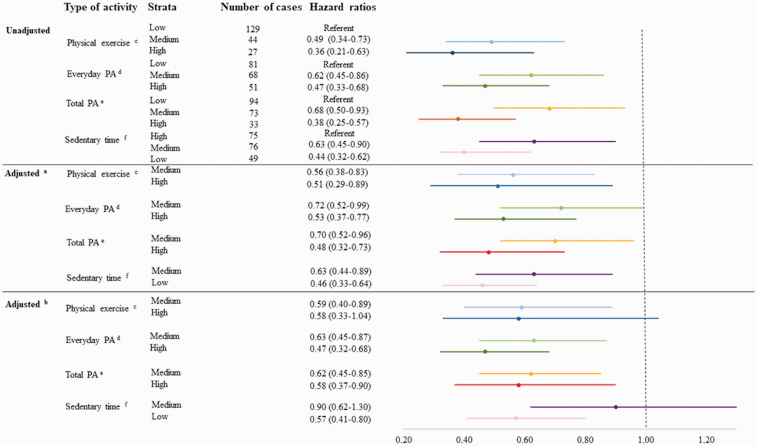
Number of cases and hazard ratios (hazard ratio with 95% confidence intervals) for mortality (200 deaths) among patients (*N* = 1148) with different physical activity levels treated on a cardiac ward. ^a^Adjusted for age and gender. ^b^Adjusted for age, disposable income, eating habits, educational level, gender, group of diagnosis, hazardous use of alcohol and smoking status. ^c^Physical exercise in an average week: low (0 min), medium (<60 min), high (≥60 min). ^d^Everyday physical activity in an average week: low (≤29 min), medium (30–149 min), high (≥150 min). ^e^Index of total physical activity level. Answers from the physical exercise and everyday physical activity question formed an index (3–19 points) of total physical activity level an average week: low (3–6 points), medium (7–9 points) and high (≥10). ^f^Sedentary time, an average day: high (≥10 h), medium (7–9 h), low (0–6 h). PA: physical activity

Baseline differences between survivors and non-survivors are described in Table 1. Median follow-up time (i.e. time between the end of treatment on the cardiac ward and date of death or end of study) was 942 days. A total of 200 deaths occurred during the study period. Total risk time was 2962 person-years with an incidence was 68 cases per 1000 person-years. Number of cases varied between different physical activity and sedentary groups (Figure 3). Hazard ratios of mortality were lower for individuals with high and medium physical activity level compared with those with low levels of everyday physical activity in the unadjusted and adjusted analyses. Compared with low physical exercise, mortality risk was lower in the medium and high physical exercise groups in the unadjusted analyses. The decreased risk remained in the adjusted analyses, except in the fully adjusted model for individuals with a high level of physical exercise (Figure 3). Further, for total physical activity level, the risk was lower for individuals reporting medium or high physical activity level compared with low in both unadjusted and adjusted models. Moreover, individuals with a low and medium level of SED had a lower hazard ratio compared to the group with high SED, except for those individuals categorized as exhibiting a medium level of SED in the fully adjusted model (Figure 3). On looking at the risk of mortality between the diagnosis groups, no difference across physical activity and SED was found.

## Discussion

The main finding of this study is that self-reported physical activity level and SED of patients prior to admission to a cardiac ward are clinically relevant predictors of hospital utilization and all-cause mortality. This is true even for patients with different diagnoses, IHD, heart failure, cardiac arrhythmias, valvular heart disease, inflammatory heart diseases and others. This highlights the importance of assessing physical activity levels and SED among patients treated on cardiac wards. Interestingly, everyday physical activity is a better predictor of readmission than physical exercise.

We demonstrated a dose–response relationship between physical activity level, SED and hospital duration. The shortest inpatient duration was seen among individuals exhibiting the highest physical activity level or lowest SED. Previous studies that have focused on the association between physical activity level and healthcare utilization in general concluded that individuals with the lowest physical activity level have the highest risk of being hospitalized^8^,^9^ and longer inpatient duration.^8^ The median inpatient duration in this study population was two (3 IQR) days on a cardiac ward. One higher step in the self-assessed physical activity level, or lower SED level, was related to an approximately 0.9 days shorter hospital stay. Inpatient duration in the present study was lower compared with the average hospital duration (4.61 days) for patients with a similar diagnosis in Sweden (in 2016).^28^ Based on our results, a change of one category of physical activity level or SED could hypothetically decrease the total hospital duration among patients with CVD in Sweden by nearly 20%. The estimation of how changes in physical activity level can reduce inpatient duration is supported by a Canadian study of healthy elderly.^29^

The present study also highlights self-reported physical activity level and SED prior to admission as predictors of future readmission. Patients with a higher physical activity level and lower SED had a decreased risk of readmission. This is in line with a study focusing on physical activity levels post-myocardial infarction.^14^

Patients not having a low level of physical activity nor a high level of SED exhibited the lowest mortality. To our knowledge there are no studies using physical activity level and SED prior to admission as predictors of all-cause mortality among patients with CVD. Interestingly, there were no differences between individuals reporting a moderate or high level of physical activity or a medium or low level of SED. Indeed, this supports the results of previous epidemiological studies^4^,^5^ illustrating that ‘a little activity is better than nothing’ and that most health benefits would be achieved by increasing physical activity among the most inactive patients with CVD.

An important novelty of the present study is that it includes patients with different CVD diagnoses. Previous studies have mainly focused on patients with heart failure or IHD.^13–15^,^30^,^31^ Our results indicate that other diagnoses such as cardiac arrhythmias, valvular heart disease and inflammatory heart diseases show a similar association of physical activity level and SED to hospital utilization and mortality. Additionally, this study includes a fairly large sample (response rate 89%). Nevertheless, the size of the study population and number of cases made it difficult to prove that the results can be applied to different age and diagnosis groups. The eight-month inclusion period was chosen to represent normal clinical circumstances. However, it did not include patients treated at weekends or overnight.

There are several limitations to this study. This study explores the association of physical activity level and SED with hospital utilization and mortality without giving information about the causality. This implies a risk of reverse causality. For example, low self-rated physical activity levels and high levels of SED prior to admission to a cardiac ward might correspond, at least partially, to the severity of the condition. However, no significant difference was seen between the different CVD diagnoses with varying severity and prognosis. In order to reduce the risk of other factors affecting the association, we adjusted for several known covariates.^21^,^22^,^32^ However, there is always the possibility that other risk factors, not available in this study, could have had an impact on the results, such as residual confounding.

A further limitation is that this study does not give any information about previous CVD, comorbidity, the treatment given during inpatient care, medication or changes in lifestyle habits post discharge. Previous studies indicate that changes in physical activity levels post myocardial infarction may affect the risk of mortality. Patients who increased their activity level had a lower mortality risk compared with those who continued to be inactive or decreased their activity level.^30^,^31^ Future studies are thus needed to investigate whether changes in physical activity level or SED between pre and post hospitalization affect hospital utilization or the risk of mortality.

One strength with the present study is that it includes physical exercise, everyday physical activity and SED, and shows that all these variables were strong predictors of hospital utilization and mortality. In the included study population, most patients participated in everyday physical activity, which is in line with previous studies on the elderly.^32^ This is an important addition, considering that previous studies have mainly focused on physical activity at moderate to vigorous intensities within exercise-based cardiac rehabilitation.^13^,^33^

Another limitation with the present study is that the physical activity level and SED are based on self-rated data. Although the questions were previously validated,^17^,^18^ there is a risk of recall bias and overestimation due to difficulties in estimating physical activity duration and intensity, and interpreting the questions, as well as social desirability.^34^ However, objective measurement of previous physical activity levels and SED is not possible. Nevertheless, the predictive validity for mortality and hospital utilization was shown to be strong, and self-reported physical activity levels may be an important measure for diagnostic purposes, containing important prognostic information. This is an important addition to previous recommendations,^6^ emphasizing the use of measuring blood pressure, blood lipids and asking questions about smoking habits as prognostic factors. The present study underlines the importance of creating a routine, also for asking patients about their physical activity level and SED in order to predict risk of future morbidity and mortality. In this context, physical activity level can be seen as an additional marker of disease severity.

In conclusion, this study found that having a low level of regular physical activity or a high level of SED, that is, being sedentary and/or physically inactive, is associated with the greatest risk in hospital utilization and all-cause mortality. The clinical impact of this is clear. First, asking all patients on a cardiac ward to self-rate their physical activity level and SED prior to admission will identify individuals in need of behavioural change. Second, by identifying and supporting those individuals who need to increase their physical activity level, clinicians might potentially decrease the utilization of inpatient care and potentially lower the risk of all-cause mortality among individuals with different CVD diagnoses. However, this needs to be shown in a prospective study. Information on physical activity level is readily available but not used uniformly, which underlines the need for interventions focusing on assessing and promoting physical activity among all patients treated on cardiac wards.

## Implications for practice

- Preceding physical activity level and sedentary time predict hospital utilization and mortality.
- The greatest health benefits will be achieved by increasing physical activity behaviour among the most inactive patients.
- Physical activity level and sedentary time should be assessed on admission to the cardiac ward, in order to identify inactive individuals.

## Supplemental Material

sj-pdf-1-cnu-10.1177_1474515120921986 - Supplemental material for Subjective reports of physical activity levels and sedentary time prior to hospital admission can predict utilization of hospital care and all-cause mortality among patients with cardiovascular disease Click here for additional data file. Supplemental material, sj-pdf-1-cnu-10.1177_1474515120921986 for Subjective reports of physical activity levels and sedentary time prior to hospital admission can predict utilization of hospital care and all-cause mortality among patients with cardiovascular disease by Amanda Ek, Lena V Kallings, Mattias Ekström, Mats Börjesson and Örjan Ekblom in European Journal of Cardiovascular Nursing

sj-pdf-2-cnu-10.1177_1474515120921986 - Supplemental material for Subjective reports of physical activity levels and sedentary time prior to hospital admission can predict utilization of hospital care and all-cause mortality among patients with cardiovascular disease Click here for additional data file. Supplemental material, sj-pdf-2-cnu-10.1177_1474515120921986 for Subjective reports of physical activity levels and sedentary time prior to hospital admission can predict utilization of hospital care and all-cause mortality among patients with cardiovascular disease by Amanda Ek, Lena V Kallings, Mattias Ekström, Mats Börjesson and Örjan Ekblom in European Journal of Cardiovascular Nursing
