# Supplementary material for: Subjective reports of physical activity levels and sedentary time prior to hospital admission can predict utilization of hospital care and all-cause mortality among patients with cardiovascular disease
Source: Eur J Cardiovasc Nurs. 2020 May 5;19(8):691–701. doi: 10.1177/1474515120921986 (PMC7817990; doi:10.1177/1474515120921986)
Supplement: sj-pdf-1-cnu-10.1177_1474515120921986 - Supplemental material for Subjective reports of physical activity levels and sedentary time prior to hospital admission can predict utilization of hospital care and all-cause mortality among patients with cardiovascular disease [file sj-pdf-1-cnu-10.1177_1474515120921986.pdf]

Questionnaire

A few questions about your lifestyle habits

Physical activity

1. **During a regular week, how much time do you spend exercising on a level that makes you short winded, for example running, fitness class, or ball games**

□ No time at all

□ ≤ 29 minutes

□ 30–59 minutes

□ 60–89 minutes

□ 90–119 minutes

□ ≥ 120 minutes

2. **During a regular week, how much time are you physically active in ways that are not exercise, for example walks, bicycling, or gardening? Add together all activities lasting at least 10 min**

□ No time at all

□ ≤ 29 minutes

□ 30–59 minutes

□ 60–89 minutes

□ 90–149 minutes

□ 150–299 minutes

□ ≥ 300 minutes

3. **How much time do you sit during a normal day, excluding sleep?**

□ Virtually all day

□ 13– 15 hours

□ 10–12 hours

□ 7–9 hours

□ 4–6 hours

□ 1–3 hours

□ Never

Eating habits

1. How often do you eat vegetables and/or root vegetables (fresh, frozen or cooked)?

□ Twice a day or more often

□ Once a day

□ Several times a week

□ Once a week or less often

2. How often do you eat fruit and/or berries (fresh, frozen, canned, juice etc)?

□ Twice a day or more often

□ Once a day

□ Several times a week

□ Once a week or less often

3. How often do you eat fish or shellfish as the main meal, in a salad or as a sandwich?

□ Three times a week or more often

□ Twice a week

□ Once a week

□ Several times a month or less often

4. How often do you eat buns and cakes, chocolate/sweets, potato crisps or carbonated/soft drinks?

□ Twice a day or more often

□ Once a day

□ Several times a week

□ Once a week or less often

Tobacco

1. My smoking habits

□ I have never been a smoker

□ I stopped smoking more than 6 months ago

□ I stopped smoking less than 6 months ago

□ I smoke, but not every day

□ I smoke every day

□ I smoke……. cigarettes per day

Alcohol

How many alcohol units do you drink in an average week?

□ Less than 1 alcohol units per week or none at all

□ 1–4 alcohol units per week

□ 5–9 alcohol units per week

□ 10–14 alcohol units per week

□ 15 or more alcohol units per week
