# Supplementary material for: Subjective reports of physical activity levels and sedentary time prior to hospital admission can predict utilization of hospital care and all-cause mortality among patients with cardiovascular disease
Source: Eur J Cardiovasc Nurs. 2020 May 5;19(8):691–701. doi: 10.1177/1474515120921986 (PMC7817990; doi:10.1177/1474515120921986)
Supplement: sj-pdf-2-cnu-10.1177_1474515120921986 - Supplemental material for Subjective reports of physical activity levels and sedentary time prior to hospital admission can predict utilization of hospital care and all-cause mortality among patients with cardiovascular disease [file sj-pdf-2-cnu-10.1177_1474515120921986.pdf]

Supplementary 2. Correlations between PA level, SED and the other covariates with inpatient duration

	B	Std. Error	Sig.
Physical exercise			
(Constant)	0,831	0,203	< 0,001
Physical exercise	-0,085	0,03	0,005
Gender	-0,058	0,047	0,217
Age	0,007	0,002	< 0,001
Diagnose	-0,029	0,013	0,025
Eating habits	0,001	0,035	0,974
Education level	-0,023	0,029	0,435
Smoking	-0,013	0,032	0,696
Hazardous use of alcohol	-0,053	0,057	0,351
Everyday physical activity			
(Constant)	0,863	0,202	< 0,001
Everyday physical activity	-0,102	0,03	0,001
Gender	-0,042	0,046	0,364
Age	0,007	0,002	< 0,001
Diagnose	-0,03	0,013	0,019
Eating habits	0,002	0,035	0,946
Education level	-0,026	0,029	0,365
Smoking	-0,011	0,032	0,742
Hazardous use of alcohol	-0,058	0,057	0,306
Total activity level			
(Constant)	0,89	0,203	< 0,001
Total activity level	-0,101	0,028	< 0,001
Gender	-0,056	0,046	0,224
Age	0,007	0,002	< 0,001
Diagnose	-0,03	0,013	0,021
Eating habits	0,008	0,035	0,817
Education level	-0,022	0,029	0,45
Smoking	-0,016	0,032	0,609
Hazardous use of alcohol	-0,057	0,057	0,315
Sedentary time			
(Constant)	0,817	0,199	< 0,001
Sedentary time	-0,082	0,026	0,001
Gender	-0,033	0,046	0,48
Age	0,008	0,002	< 0,001
Diagnose	-0,031	0,013	0,019
Eating habits	-0,004	0,035	0,907
Education level	-0,034	0,029	0,24
Smoking	-0,004	0,032	0,888
Hazardous use of alcohol	-0,061	0,057	0,285

Note: Residuals of the natural logarithm (ln) of inpatient duration was used due to being normally distributed.
